# Cannabinoids in traumatic brain injury and related neuropathologies: preclinical and clinical research on endogenous, plant-derived, and synthetic compounds

**DOI:** 10.1186/s12974-023-02734-9

**Published:** 2023-03-19

**Authors:** Brittney R. Lins, Chidozie C. Anyaegbu, Sarah C. Hellewell, Melissa Papini, Terence McGonigle, Luca De Prato, Matthew Shales, Melinda Fitzgerald

**Affiliations:** 1 Curtin Health Innovation Research Institute, Curtin University, Bentley, 6102 Australia; 2 Perron Institute for Neurological and Translational Science, Nedlands, 6009 Australia; 3 MediCann Health Aust Pty Ltd, Osborne Park, 6017 Australia

**Keywords:** Traumatic brain injury, Concussion, CNS injury, Neurotrauma, Endocannabinoid system, Terpenes, Neuroprotection, Inflammation

## Abstract

Traumatic brain injury is common, and often results in debilitating consequences. Even mild traumatic brain injury leaves approximately 20% of patients with symptoms that persist for months. Despite great clinical need there are currently no approved pharmaceutical interventions that improve outcomes after traumatic brain injury. Increased understanding of the endocannabinoid system in health and disease has accompanied growing evidence for therapeutic benefits of *Cannabis sativa*. This has driven research of Cannabis’ active chemical constituents (phytocannabinoids), alongside endogenous and synthetic counterparts, collectively known as cannabinoids. Also of therapeutic interest are other Cannabis constituents, such as terpenes. Cannabinoids interact with neurons, microglia, and astrocytes, and exert anti-inflammatory and neuroprotective effects which are highly desirable for the management of traumatic brain injury. In this review, we comprehensively appraised the relevant scientific literature, where major and minor phytocannabinoids, terpenes, synthetic cannabinoids, and endogenous cannabinoids were assessed in TBI, or other neurological conditions with pathology and symptomology relevant to TBI, as well as recent studies in preclinical TBI models and clinical TBI populations.

## Traumatic brain injury

Traumatic brain injury (TBI) is a common event associated with motor vehicle accidents, sports, assaults, and intimate partner violence [1–3]. TBI is caused by an impact and/or rotational force applied to the head which may cause alteration in consciousness alongside a range of symptoms in physical, cognitive, emotional or sleep-related domains [4–6]. TBI occurs on a severity spectrum of mild to severe injury. Those at the severe end of this spectrum often experience substantial impairment which may impede ability to work or study; reduce quality of life; and incur significant financial burden [7–9]. While the majority of TBIs are mild (mTBI, also known as concussion), 10–20% of mTBI patients experience persistent neurological deficits for months or years in a clinical presentation called persisting post-concussive symptoms [10–12]. The long-term outcomes of repeated mTBI, such as may occur over an athletic career, have been reported to include risk of cognitive impairment, depression, and motor deficits persisting years to decades post-injury [12–16], although the quality of evidence is such that further work is needed. Repeated mTBI is also associated with chronic traumatic encephalopathy, a neurodegenerative disorder which relies on post-mortem diagnosis but may share clinical signs and symptoms with Alzheimer’s and Parkinson’s diseases [17]. These clinical findings have also been observed in animal studies, with repeated mTBI associated with worse outcomes in a variety of preclinical models [18–23]. Despite concerted efforts, the only treatment options for TBI include symptom management and cognitive therapies [24]. There are currently no effective pharmaceutical treatment options to improve outcomes for TBI of any severity despite great clinical need.

The field of cannabinoid research has developed rapidly, and the rationale for use of cannabinoids in the management of neurological conditions such as TBI is increasingly recognized. This review provides a brief overview of the endocannabinoid system in the central nervous system (CNS); introduces cannabinoids; and summarizes the putative benefits of endogenous, plant-derived, and synthetic compounds in TBI models, or models with pathology relevant to TBI. Finally, clinical studies on phytocannabinoids and synthetic cannabinoids in TBI are reviewed.

## The endocannabinoid system

The major constituents of the endocannabinoid system are endogenous neurotransmitters collectively known as endocannabinoids, and two key cannabinoid receptors. The two most well-characterized endocannabinoids are N-arachidonylethanolamine (also known as anandamide; AEA) and 2-arachydonoyl glycerol (2-AG) [25], although the endocannabinoid system continues to expand as research progresses [26]. The two key endocannabinoid receptors are cannabinoid receptor 1 (CB1R) and cannabinoid receptor 2 (CB2R) [27, 28]. Both CB1R and CB2R are G-protein coupled receptors that, when activated, inhibit voltage-gated N-type and P/Q-type Ca^2+^ channels [29]. In the CNS, CB1R is expressed on neurons at the presynaptic terminal and on astrocytes, while CB2R is expressed on microglia, the resident macrophage/monocyte immune cells of the brain [28, 30]. In addition to these two major cannabinoid receptors (CB1R and CB2R), other receptors are involved in the endocannabinoid system, with the extent and significance of their roles still emerging (recently reviewed by Cristino and colleagues) [26]. The most notable of these additional receptors are: transient receptor potential vanilloid-1 (TRPV1), various G-protein-coupled receptors (GPR55, GPR13, GPR6, GPR12, GPR18), peroxisome proliferator-activated receptor-γ (PPARγ), serotonin receptors (5-HT_1A_), and adenosine receptors (A_2A_) [26, 28]. Of these, TRPV1 and PPARγ are most likely of relevance to TBI treatment due to the known roles of TRPV1 in pain management, and PPARγ in inflammation and neurodegeneration [31, 32].

A well-established role of the endocannabinoid system in the CNS is the suppression of both excitatory and inhibitory signaling in an activity-dependent manner, an effect mediated by AEA and 2-AG binding to CB1R in neurons [33–36]. The production of AEA and 2-AG is initiated by increased cellular firing rates and the associated elevation of intracellular Ca^2+^ levels within the postsynaptic neuron [34, 36]. AEA and 2-AG are then released from the postsynaptic cell membrane to bind CB1R on the presynaptic membrane, subsequently blocking Ca^2+^ channels to inhibit further neurotransmitter release. This is known as inhibitory retrograde neuromodulation [26, 36]. The ‘on-demand’ production of endocannabinoids allows them to act as a negative feedback mechanism in response to high levels of neural activity, a phenomenon known as depolarization-induced suppression of excitation, or depolarization-induced suppression of inhibition, depending on whether the presynaptic neuron is excitatory or inhibitory, respectively [33]. This is relevant to TBI, where increased cellular firing and excitotoxicity are prominent pathological events, and suppression of these effects may be neuroprotective [37].

Another important role of the endocannabinoid system in the CNS relates to participation in an endogenous injury response system mediated by CB2R located on microglia [38]. This is evidenced by increased upregulation of the CB2R in injured brain parenchyma in the hours and days following injury [38]. Endocannabinoid concentrations are also elevated after injury, with increased 2-AG hypothesized to protect the blood–brain barrier (BBB) and inhibit the expression of pro-inflammatory cytokines, regulating the inflammatory response [39, 40]. As a whole, emerging evidence supports the role of the endocannabinoid system as a compensatory, neuroprotective, injury-response system after TBI. Manipulation of this system through administration of exogenous compounds or modulation of endogenous factors is emerging as an attractive therapeutic strategy for TBI.

## Cannabinoids and related compounds

In addition to the endogenous neurotransmitters produced in the mammalian nervous system (AEA and 2-AG, described above), exogenous compounds also influence the endocannabinoid system. Collectively, these are known as cannabinoids and they may be endogenous (endocannabinoids), derived from the Cannabis plant (phytocannabinoids), or synthetic (synthetic cannabinoids).

### Phytocannabinoids and terpenes

Phytocannabinoids are naturally occurring compounds isolated from the Cannabis sativa plant with biological activity within the endocannabinoid system [41]. The major phytocannabinoids are ∆9-tetrahydrocannabinol (THC) and cannabidiol (CBD).

THC is the most well-known phytocannabinoid and the main psychoactive constituent of the cannabis plant [35]. The pharmacological activity of THC is similar to that of endocannabinoid AEA, whereby it acts as a partial agonist at CB1R and CB2R [29, 35, 42]. In addition, similar to AEA, THC administration suppresses neuronal firing at the presynaptic level [43, 44]. Notably, two synthetic THC compounds are approved by the FDA for medical use in the United States: nabilone (marketed as Cesamet, a synthetic THC derivative) and dronabinol (marketed as Marinol, synthetic THC). Both are indicated for chemotherapy-induced nausea and vomiting, with dronabinol also used to treat anorexia in patients with AIDS [29, 45–47]. Although it is possible that THC could limit nausea and vomiting within the context of acute TBI, THC has also been directly studied in models of TBI pathology and indirectly in some clinical TBI populations with encouraging results for therapeutic potential beyond the context of nausea (refer to Tables 2 and 4).

The other major cannabis constituent, CBD, possesses complex pharmacology which continues to be the focus of emerging research. CBD is generally regarded as an antagonist of CB1R and an inverse agonist of CB2R [35, 48], although other reports describe CBD as a negative allosteric modulator of CB1R [49, 50]. CBD also potentiates the effects of endocannabinoid AEA by inhibiting fatty acid amide hydrolase (FAAH), the enzyme responsible for AEA degradation [29]. This is relevant to TBI, because FAAH inhibitors restore BBB integrity and improve motor, cognitive and mood-related outcomes in models of TBI [51–53]. In addition, a variety of other receptor–ligand interactions within the extended endocannabinoid system have been characterized [29]. CBD is a direct agonist at serotonin 5-HT_1A_ receptors (with implications for mood and anxiety-like effects), a weak agonist at TRPV1 receptors (with implications for pain and synaptic plasticity), a PPARγ agonist (with implications for neurodegeneration and inflammation), and an indirect agonist at adenosine (A_2A_) receptors [28, 42]. The effects of CBD at these receptors may confer benefits in TBI, particularly PPARγ activation which reduces neurodegeneration and inflammation [31].

While THC and CBD are the most abundant and well-studied phytocannabinoids, Cannabis sativa contains over 140 minor phytocannabinoids and other pharmacologically active compounds [29, 42]. These minor phytocannabinoids are naturally present in the Cannabis plant, and understanding their effects is integral to realizing the therapeutic potential of Cannabis, as well as to guide the utilization of full spectrum, plant-derived treatments, or design compound blends for medicinal purposes. While research on minor cannabinoids has increased, there is still very little known regarding their effects in normal physiology and disease, including TBI.

In addition to phytocannabinoids, the cannabis plant contains compounds known as terpenes, some of which also possess biological activity with potential therapeutic benefits [54–56]. Terpenes are naturally occurring, potent hydrocarbons found in a variety of botanical sources, including Cannabis. They are responsible for the odors and flavors associated with Cannabis and various terpenes are used as food additives that are generally recognized as safe by the US Food and Drug Administration [54]. Terpenes commonly associated with Cannabis include β-caryophyllene, myrcene, limonene, linalool, terpineol, γ-terpinene, α-pinene, β-pinene, nerolidol, phytol, and citral, although this is not an exhaustive list [54–56]. The role that terpenes play in the medicinal effects of cannabis, and how these interact with other biologically active plant constituents is of interest for optimizing their therapeutic potential [56].

Cannabis plant-derived extracts have proven attractive options for therapeutic development, with two botanically derived cannabis products approved for medical use by the Federal Drug Administration in the United States. These are Sativex^®^, a blend of high THC and high CBD plant extracts in a 1:1 ratio for the treatment of pain and spasticity in Multiple Sclerosis, and Epidiolex^®^, a pure CBD extract for severe forms of epilepsy [33]. The clinical success of Epidiolex and Sativex are encouraging for future application of cannabis extracts in other conditions, such as TBI.

### Synthetic cannabinoids

In addition to endogenous and plant derived compounds, synthetic cannabinoids are not naturally occurring, but rather produced through lab-based synthesis. Synthetic cannabinoids may be derived from naturally occurring cannabinoids; interact with endogenous cannabinoid receptors; or otherwise resemble endocannabinoids and phytocannabinoids [41]. Synthetic cannabinoids may differ from their endogenous and plant-derived counterparts by affecting distinct receptors, or by achieving different levels of potency or binding specificity than naturally occurring cannabinoids [57, 58]. To summarize, cannabinoids are a diverse class of molecules produced through natural and synthetic means which possess biological activity within the endocannabinoid system.

## Preclinical research of cannabinoids in tbi and related models

Several endogenous, plant-derived, and synthetic cannabinoids have been explored in a variety of preclinical TBI and TBI-related models, such as spinal cord injury and stroke, and these are reviewed below. Additional compounds that have not been assessed directly in TBI, but have displayed promising effects in TBI-relevant pathologies are also reviewed.

### Endocannabinoids in TBI and related conditions

The neuroprotective role of endocannabinoids and the endocannabinoid system in TBI is supported by emerging preclinical research in a variety of TBI models (Table 1). For example, in a mouse model of closed-head TBI, synthetic 2-AG administration reduced edema and infarct volume; mitigated cell death in the hippocampus; and improved functional recovery. These effects were CB1R-dependent, supporting a protective role for the endocannabinoid system in response to injury [39]. These neuroprotective effects were further demonstrated in a study which potentiated 2-AG effects through inhibition of its degrading enzyme monoacylglycerol lipase (MAGL) in the controlled cortical impact (CCI) model [59]. In this study, administration of the MAGL inhibitor MJN110 in CCI mice after injury reduced inflammatory markers; attenuated cell death; restored glutamate and GABA receptors changes; and improved cognitive and locomotor behaviors [59]. In a repeated mTBI mouse model, knockout of astrocyte-specific MAGL reduced neuroinflammation; attenuated TBI-induced gene expression changes; and prevented neurodegenerative pathology and cognitive impairment, among other injury-induced effects. Again, these benefits were mediated by CB1R [60]. Similarly, administration of FAAH inhibitor URB597 was neuroprotective in an oxygen–glucose deprivation model of injury, with anti-oxidant, anti-inflammatory, and anti-apoptotic effects [61]. Finally, in a model of kainic acid-induced excitotoxicity, AEA rapidly increased in the hippocampus, conferring neuroprotection in a CB1R-dependent manner [34, 40]. These findings support the neuroprotective effects of the endocannabinoid system in response to injury, and demonstrate that endocannabinoid augmentation or supplementation may have therapeutic benefits in TBI.

**Table 1:** Endocannabinoids and endocannabinoid modulation in TBI models

Treatment	Dose	Route	Frequency	Model	Species	Results	References
Synthetic 2-AG administration	0.1, 5, 10 mg/kg	i.v.	15 min post-injury	Weight-drop TBI (closed-head)	Sabra mice, males	Treatment educed edema; reduced infarct volume; reduced cell death (hippocampus); and improved functional recovery	[39]
2-AG augmentation (via MAGL inhibitor MJN110)	0.5, 1, 2.5 mg/kg	i.p.	30 min after each injury, then daily for 5 days (8 × total)	Repetitive closed-head injury (CCI device); three impacts delivered 24 h apart	C57BL/6 mice, males	Treatment reduced inflammatory markers; attenuated cell death; restored glutamate and GABA receptors changes; improved cognition; and improved locomotor function	[59]
2-AG augmentation (via astrocyte-specific MAGL knockout)	n/a	n/a	n/a	Repetitive closed-head injury (CCI device); three impacts delivered 24 h apart	Mgll^flox/flox^ mice (astrocyte-specific MAGL knockout), males and females	Knockout reduced neuroinflammation; reduced TBI-induced gene expression changes; and prevented neurodegenerative pathology and cognitive impairment, among other injury-induced effects	[60]

### Major phytocannabinoids in TBI models

Few preclinical studies have directly assessed the major constituent phytocannabinoids in models of TBI, although the available data are encouraging (Table 2). In a weight-drop mTBI mouse model, injury resulted in impaired sociability, heightened aggression, and tactile allodynia 14 days later, all of which were improved by daily oral treatment with 10% CBD oil for either 14 days, or from days 50–60 post-injury [62]. In this same study, mTBI increased levels of D-aspartate, glutamate and GABA in the medial prefrontal cortex, and CBD treatment ameliorated these changes [62]. In a rat weight-drop with craniotomy model of TBI (modified Feeney’s model), CBD (10 mg/kg) was administered both 30 min before injury and 6 h post-injury [63]. The authors demonstrated beneficial effects on BBB integrity, whereby CBD treatment reduced aquaporin-4 expression and increased expression of claudin-5 and occludin, with BBB leakage directly observed using Evan’s Blue assay. CBD treatment was also found to decrease GFAP expression, and reduced concentrations of the proinflammatory mediators TNF-α and IL-1β compared to control [63]. In rats with moderate TBI (CCI model), Friedman and colleagues [64] applied several high CBD, low THC botanical preparations directly to the open skull, over the dura above the injury site via a gelfoam matrix after injury. Additional groups received the cannabinoid gelfoam matrix as well as systemic administration of CBD 10 min after injury, and on 14 non-consecutive days thereafter [64]. The combination of gelfoam and systemic injection was more effective than gelfoam or systemic administration alone, with decreased defecation scores (an anxiety-like measure), smaller lesion volumes, reduced hippocampal neuron loss and neural pathology [64]. This combination treatment also had anti-inflammatory effects, indicated by reduced GFAP immunoreactivity, with concomitant improvements in motor and cognitive function [64]. While the gelfoam preparation may not be suitable for all types of TBI, it may be a useful strategy in the context of penetrating head wounds [64]. In conclusion, these studies demonstrate increasing evidence that CBD treatment is multifunctional, with beneficial effects on inflammation, cognition, neurobehavioral deficits, lesion volumes and BBB breach after TBI.

**Table 2:** Phytocannabinoids in TBI models

Treatment	Dose	Route	Frequency	Model	Species	Results	References
THC	1.25 mg/kg	i.p.	Daily ×12, post-injury or 6× intermittent treatments prior to injury	Closed-head lateral impact 3×, 3 days apart (rmTBI)	Sprague–Dawley rats, males and females	Post-rmTBI treatment reduced anxiety behavior in the elevated plus maze and prevented telomere shortening. Effects on depression-like outcomes were sex-dependent	[65]
THC	1 mg/kg	i.p.	1×, 1 h post-injury	Closed-head, weight-drop with rotation (mTBI)	Sprague–Dawley rats, males and females	THC did not affect TBI-induced RotaRod impairment (males); THC impaired RotaRod performance in sham rats (males); THC treatment increased IL-6 after TBI (males); and THC + TBI reduced CB1R density relative to THC alone (females)	[67]
THC	3 mg/kg	i.p.	Daily ×3, post-injury	CCI (with craniotomy)	C57BL/6 J mice, males	Treatment improved RotaRod performance; and upregulated G-CSF, BDNF, GDNF	[68]
THC	3 mg/kg	i.p.	Daily ×3, post-injury	CCI (with craniotomy)	C57BL/6 J mice, males	Treatment reversed CCI-induced impairment in spontaneous alternation Y-maze performance; improved RotaRod performance; and upregulated 2-AG, G-CSF, BDNF, GDNF	[69]
CBD	30 μL, oil 10%	Oral gavage	Daily from days 1–14 and days 50–60 post-injury	Weight-drop mTBI with longitudinal incision	C57BL/6 J mice, males	Treatment reduced pain response; improved sociability; reduced aggression; and restored levels of D-Asp, Glutamate and GABA 14 days after mTBI, but not 60 days after mTBI	[62]
CBD	5, 10, 20 mg/kg	i.p.	30 min before and six h post-injury	Weight-drop TBI with craniotomy (modified Feeney’s model)	Sprague–Dawley rats, males	Treatment (10 mg/kg) downregulated TNF-α, IL-1β, GFAP, AQP4; upregulated claudin-5 and occludin; and reduced edema	[63]
High CBD, low THC preparation	20 mg/mL topical; 20–40 mg/kg	Topical via gel, ± i.p.	Gel applied 1× two min post-injury; i.p. administered 10 min post-injury then daily × 14	CCI (with craniotomy)	Sprague–Dawley and Wistar rats, males and females	Treatment improved vestibulomotor function; improved cognitive function; reduced lesion volume; and attenuated hippocampal injury	[64]

The available data on THC treatment in TBI models are more nuanced than that of CBD, with sex and timing emerging as important variables. In a repeated injury weight-drop model in rats, THC (1.25 mg/kg, i.p.) was administered as six intermittent pre-injury treatments or 12 consecutive post-injury treatments. The post-injury THC treatment improved anxiety-like behavior in the elevated plus maze, but not in the open field task [65]. Depression-like behavior in the forced swim task was also improved in male rats treated post-injury with THC, although a more severe depression-like phenotype was seen in females [65]. In the same study, THC treatment post-injury also prevented telomere shortening after repeated mTBI [65]. Consistent with prior studies [22, 66], the authors found that repeated mTBI increased microglial activation. However, they did not observe an effect of THC treatment on microglial activation in limbic system structures (the hippocampus and nucleus accumbens). Rather, THC increased IBA1 immunoreactivity in the prefrontal cortex, implying THC may not have a therapeutic effect on this measure of neuroinflammation [65]. In a similar rat model with a single weight-drop mTBI, a single injection of THC (1 mg/kg, i.p.) did not improve TBI-induced deficits in motor function, and treatment impaired motor function in sham-injured controls, though this was only seen in males [67]. Other sex-specific effects of THC included increased levels of cytokine interleukin-6 after TBI in males only, while female TBI rats that received THC had a reduced density of CB1R compared to those that received THC, but no injury [67]. This indicates that THC effects are sex-specific in both uninjured and TBI conditions, with variable effects on behavior outcomes, inflammatory responses, and alterations to the endocannabinoid system [67]. In contrast, in a mouse CCI model of TBI that assessed males only, THC treatment (3 mg/kg, i.p., daily for 3 days) improved motor function in the RotaRod test, although this difference may be due to dose and treatment frequency as both were greater than the previously mentioned study in rats. This improved motor performance was accompanied by upregulated brain-derived neurotrophic factor and glial-derived neurotrophic factor, which are associated with neuronal and glial repair, respectively, in different brain regions [68]. THC treatment was also associated with increased 2-AG levels in the brain and improved short term working memory in the spontaneous alternation Y-maze test [69]. These studies are encouraging for the potential application of THC in the management of TBI, though further research is necessary to investigate appropriate dosing regimens and determine sex-specific effects, particularly with regard to neuroinflammation.

### Major phytocannabinoids in TBI-related conditions

In addition to the limited studies that directly assessed TBI models, models of CNS injury with related pathology such as spinal cord injury (SCI) and stroke further support the therapeutic potential of phytocannabinoid treatment in neurotrauma. In a spinal cord contusion model, CBD treatment (1.5 mg/kg; i.p.) was administered repeatedly post-injury 1 h, 24 h, and 3 days later, then continued twice per week until the end of the experiment [70]. This treatment reduced expression of inflammatory cell markers in the spinal cord, and improved thermal sensitivity after SCI. However, locomotor and bladder function were not affected by CBD treatment [70]. CBD has also been assessed in stroke models, such as the carotid artery occlusion model. CBD (10 mg/kg) administered 30 min before, and three, 24 and 48 h post-stroke, ameliorated anxiety-like behavior, depression-like behavior, and cognitive impairment observed in this model, as well as prevented neurodegeneration in the hippocampus and white matter loss in the corpus callosum. CBD treatment also prevented artery occlusion-induced microglial activation in several areas of the hippocampus (CA1, CA2/3, but not CA4) [71]. These studies offer additional support for phytocannabinoids as treatments for CNS injury.

### Minor phytocannabinoids in TBI-related conditions

The pharmacology of minor cannabinoids and their potential therapeutic effects is an area of active research. While much of this research is ongoing, notable minor cannabinoids with potential neuroprotective properties have been recently reviewed (see Stone et al. [88]). Notably, no studies of minor constituent cannabinoids in TBI models have been conducted to date, though effects on neurodegeneration and inflammation are relevant to TBI and in vivo studies are briefly summarized here.

∆9-Tetrahydrocannabivarin (THCV) is generally considered to have minor effects as a CB1 antagonist in vivo, and has high-affinity for CB2R, where it acts as a partial agonist, though it has recently been described as an agonist at both CB1R and CB2R [42, 72]. With this pharmacological profile, THCV could have similar effects as THC and CBD in TBI, though direct studies are required for confirmation. THCV also acts through 5-HT_1A_ receptors and shows promise as an antipsychotic and anticonvulsant [35, 54, 73]. ∆9-Tetrahydrocannabinolic acid (THCA) was beneficial in a mouse model of Huntington’s disease with effects through PPARγ activation which resulted in reduced microgliosis, astrogliosis, and dampening of pro-inflammatory markers alongside improved motor deficits [74, 75]. THCA also had anti-nociceptive, anxiolytic, and hyperlocomotive properties mediated by CB1R and CB2R agonist activity, as well as PPARγ [42].

Minor cannabinoid cannabichromene (CBC) has not been extensively tested in models of neurological disease. In vitro studies suggested CBC had pharmacological activity as a CB1R and CB2R partial agonist with greater selectivity and potency at CB2R, and also as a TRPV1 desensitizing agonist. These are promising actions for CB2R-mediated neuroprotection in conditions like TBI, as well as analgesia [42, 76, 77]. In rodents, CBC had topical and peripheral (i.e., non-CNS) anti-inflammatory effects [78–80] as well as antidepressant-like effects [81].

Cannabidiolic acid (CBDA) appears to be a partial CB2R agonist with anticonvulsant properties. Comparable effects to CBD were found in a rat maximal electroshock seizure model and a Scn1aRX/+ mouse model of Dravet syndrome hyperthermia, where treatment increased the temperature threshold required to induce tonic–clonic seizures [82, 83]. Another minor cannabinoid, cannabidivarin (CBDV) also reduced seizure activity in multiple in vivo models of epilepsy, and improved neurobehavioral abnormalities in mouse models of Rett syndrome [84, 85] Cannabinol (CBN) treatment delayed the progression of motor abnormalities in a model of amyotrophic lateral sclerosis [86].

Minor phytocannabinoid cannabigerol (CBG) is an activator of PPARγ and partial agonist of CB1R and CB2R [42]. Although CBG has not been studied in TBI models, non-cannabis derived PPARγ activator pioglitazone was neuroprotective in a rat model of TBI [87]. Pioglitazone exerted neuroprotective effects by downregulation of inflammatory NF-κB and IL-6 pathways, supporting the potential for PPARγ activators such as CBG to dampen neuroinflammation associated with TBI [87]. Despite no direct studies in TBI, CBG and its derivatives, VCE-003 and VCE-003.2, have been studied in several in vivo models of neurodegeneration and inflammation [42, 88]. In a model of Huntington’s disease, CBG derivatives improved motor performance in the RotaRod test; reduced neuron loss; enhanced neurogenesis; suppressed microglial activation and astrogliosis; mitigated the release of inflammatory enzymes and cytokines and downregulated Huntington’s disease-associated genes [89–91].

CBG derivatives have been further assessed in rodent models of multiple sclerosis, where beneficial effects on myelin and oligodendrocyte health are relevant to TBI [92–94]. CBG derivatives suppressed activation of microglia and macrophages as well as reduced levels of inflammatory mediators; preserved myelination quantity and integrity; reduced axonal damage; and improved motor outcomes [93, 94]. In the lipopolysaccharide-induced Parkinson’s disease model, CBG derivatives were neuroprotective and anti-inflammatory [95, 96]. Treatment resulted in dampened elevation of inducible nitric oxide synthase (a key mediator of inflammation); prevented elevation of pro-inflammatory cytokines TNF-α and IL-1β; reduced microgliosis; and preserved dopaminergic nigrostriatal neurons [95, 96]. Exploratory locomotor behavior was also partially improved in the cylinder rearing test [95]. In addition, in a genetic mouse model of amyotrophic lateral sclerosis, administration of a CBG derivative delayed disease progression, reduced the number of pathological signs present; and improved the clinical score while preventing weight loss [97].

The current literature on the medicinal benefits of minor cannabinoids in neurological diseases are encouraging, and further research will continue to elucidate their effects. At present, studies that directly assess minor cannabinoids in models of TBI are lacking, although the effects on overlapping pathological mechanisms described above may extend to TBI pathology. In particular, the myelin-preserving effects of CBG (and derivatives) in MS models, and reduction of microglia and astrocyte activation are candidate effects that may be relevant to TBI treatment and should be studied directly. The anti-inflammatory effects of THCV and the CB2R agonist activity of CBC are also promising and warrant further investigation.

### Terpenes in TBI-related conditions

At present, terpenes have not been directly assessed in models of TBI, though studies in other disease models with relevant pathologies can be used to infer potential benefits. A recent extensive review of terpenes by Gonçalves and colleagues [55] included discussion of their antimicrobial and anti-tumor properties, and beneficial effects in the treatment of gastrointestinal diseases, though these are largely beyond the scope of this review. Effects of terpenes on pathology with relevance to TBI (such as inflammation, oxidative stress, neurodegeneration, and pain) are summarized below.

The terpene most widely investigated in neurological conditions is β-caryophyllene (BCP) [55]. Relevant benefits of BCP included analgesic effects in mouse models of neuropathic pain and peripheral neuropathy which were blocked by co-administration of a CB2R antagonist, which suggested that these effects were mediated via the CB2R receptor [98, 99]. BCP has alsoreduced DNA oxidation and GFAP expression in a d-galactose model of aging in mice, although it failed to restore cognitive (spatial memory) deficits at the dose examined [100]. In a stroke model of bilateral common carotid artery occlusion with reperfusion, BCP modulated the response of the endocannabinoid system and prevented increased levels of lipoperoxidases, which indicated protection from oxidative damage [101]. BCP also had beneficial effects for white matter preservation, with reduced axonal demyelination and improved motor function in the experimental autoimmune encephalitis model of multiple sclerosis in mice. These effects were associated with an improved neuroinflammatory state characterized by inhibition of microglia, CD4+ and CD8+ T lymphocytes, and modulation of Th1/T_reg_ immune balance [102]. All of these effects were CB2R-dependent, and suggest neuroprotective potential. Additional studies have been conducted in non-CNS disease models, such as cancer and inflammatory bowel disease [103–107]. While beyond the scope of this review, it is notable that a mouse model of inflammatory bowel disease probed the mechanism of BCP’s anti-inflammatory effects and found that PPARγ, as well as CB2R, were required for the observed immune modulation [103]. This links BCP into the wider endocannabinoid system beyond CB2R, although further research is required to understand whether these findings translate to the CNS. Overall, numerous studies support the anti-inflammatory and analgesic effects of BCP and its pharmacological activity as an agonist at CB2R, which suggests that BCP is a worthy candidate for future research in TBI [55, 108].

An in vitro cell culture study examined the activity of ten terpenes commonly found in medical cannabis cultivars and found that the majority of the observed TRPV1-dependent Ca^2+^ response could be attributed to the terpene myrcene [109]. The effect of myrcene on TRPV1 Ca^2+^ flux was variable depending on intracellular Ca^2+^ levels, implying sensitivity to the intracellular environment [109]. Therefore, myrcene may have differential effects in health and disease states. Additional research will be needed to determine if myrcene in a high Ca^2+^ environment serves to regulate or potentiate intracellular Ca^2+^ levels. In addition, pre-application of myrcene in a cell culture model modulated CBD binding at TRPV1, suggesting myrcene may either compete with CBD, or act as an allosteric modulator [109]. These data suggest myrcene interacts with other cannabinoids and may uniquely modulate TRPV1, though further research is required to understand myrcene’s potential role in pain management [109].

Limonene is found in Cannabis as well as various citrus oils [55]. While mainly studied in terms of antifungal or antibacterial properties, limonene has also been examined in pain models, where it inhibited nociceptive behaviors [110, 111]. A high limonene content oil (not sourced from Cannabis but rather from citrus lumina) prevented downstream effects of oxidative stress such as cell death, production of reactive oxygen species (ROS), and inflammation in a drosophila model of Alzheimer’s disease [112]. Linalool had potent effects on ROS and lipid peroxidation, where it slowed cell death and improved mitochondrial morphology [113], while terpineol displayed anti-nociceptive and neuroprotective properties, as well as suppressed inflammatory cell production [55, 114]. Similarly, γ-terpinene reduced edema and inflammatory cell infiltration in mouse models of inflammation [115].

α-Pinene has various neuroprotective effects relevant to TBI. Of these, anti-oxidant activity may be most beneficial acutely after injury, with evidence that it reduced DNA damage and ROS production in skin cells after UVA light exposure [116]. Similarly, in a model of ischemic stroke, α-pinene prevented oxidative damage and inflammation, and rescued behavior deficits [117]. Nerolidol appears to have similar effects, with evidence that it prevented DNA damage through upregulation of nitric oxide levels [118]. Although these were studied in the context of parasitic infection (mice infected with Trypanosoma evansi), these mechanisms have potential relevance to TBI. Finally, citral, found in Cannabis as well as lemongrass, had anti-inflammatory properties, including inhibition of various inflammatory pathways which was partially dependent on PPARγ [119]. Citral may also affect downstream signaling from CB2R [120] and regulate cellular antioxidant defenses, such as glutathione enzymes and superoxide dismutase [121]. These findings imply citral may be beneficial in some aspects of TBI pathology.

In summary, there is a large body of evidence supporting potential therapeutic properties of terpenes, including anti-inflammatory, anti-nociceptive, and neuroprotective effects [55]. No studies have directly assessed terpenes in models of TBI, but current evidence suggests they should be considered as an integral part of the complex polypharmacy of the Cannabis plant and may contribute to the therapeutic potential of Cannabis plant extracts.

### Phytocannabinoid polypharmacy

The ‘entourage’ effect is the supposed benefit that occurs when phytocannabinoids are used in combination [54]. These benefits range from reducing or counteracting adverse effects to possible synergistic benefits. The ability to modulate potential adverse effects while maintaining or enhancing medicinal benefits would be of great value for the application of phytocannabinoids as therapeutics. The existence of the ‘entourage’ effect remains controversial, and is an area of active research, yet the potential for a tailored therapeutic benefit from cannabinoid polypharmacy presents a unique advantage that plant-based extracts may offer over isolates or synthetic compounds.

The two plant-derived pharmaceuticals with current FDA approval are Epidiolex (CDB extract) and Sativex (1:1 THC/CBD combination). While the active ingredient of Epidiolex is purified CBD [122], Sativex is a roughly 1:1 combination of a high THC and high CBD whole plant extracts, which includes minor cannabinoids [123]. The benefits of combining CBD with THC have been acknowledged in the case of Sativex, combining evidence from numerous studies in animals and humans from throughout the twentieth century, as comprehensively reviewed by Russo and Guy [124] and McPartland and colliagues [43]. Briefly, higher doses of THC are tolerated when administered with CBD, and CBD reduced adverse effects of THC, such as tachycardia, intoxication, and sedation, without reducing the beneficial effect of reduced muscle spasticity [29, 124, 125]. In addition, THC alone had a moderate and long-lasting analgesic effect that was potentiated when CBC was co-administered in an electroshock seizure model, although this synergistic effect did not extend to neurobehavioral effects (motility), anti-seizure effects, or conditioned avoidance responses [126]. The minor cannabinoid CBC has also been studied in combination with THC. DeLong and colleagues [79] demonstrated that CBC potentiated the effects of THC in the behavior tetrad (a behavior battery associated with CB1R stimulation which examines analgesia, catalepsy, locomotion, and hypothermia [127]), by achieving these effects at a lower dose than when THC was administered alone [79]. The anti-inflammatory effects of CBC were also augmented when CBC and THC were administered together [79].

Some minor phytocannabinoids appear to modulate the effects of THC. For example, a number of minor cannabinoids appear to have weak or partial agonist activity at CB1R and CB2R which, when administered alongside higher concentrations of THC, may act as functional antagonists [42]. In addition, CBD and minor constituents may modulate the effects of CB1R and CB2R activity through other ligands, though the mechanisms are not fully understood [42]. This emerging evidence supports further investigation of the interactions of cannabinoids in vivo, and the effects of full-spectrum Cannabis extracts, where the combination of various major and minor constituents may result in distinct effects compared to isolate preparations.

The terpene profile of a cannabis cultivar may also contribute to its effects, including entourage effects, although consequences of interaction with other phytocannabinoids remain largely unknown. While some studies report no effect of various terpenes on CB1R or CB2R-mediated effects [128, 129], one study demonstrated a weak association between BCP and CB2R, aligning with a previous finding that BCP is a CB2R selective agonist [99]. Despite a lack of understanding of the mechanisms by which terpenes may contribute to an entourage effect, synergy between terpenes and cannabinoids have been reported, as reviewed by Russo [54]. The functional outcomes of interactions between terpenes and cannabinoids remains an area of active research and debate, but is an important consideration in the therapeutic application of full-spectrum cannabis extracts.

### Synthetic cannabinoids in TBI

Synthetic cannabinoids have been relatively well studied in TBI models (summarized in Table 3), particularly regarding CB2R-mediated effects. In a weight-drop model in mice and rats, a single 10 mg/kg dose of CB2R agonist HU-914 or 5 mg/kg HU-910 administered 1 h after injury improved the neurological severity score compared to vehicle or lower treatment doses [130]. This benefit was completely blocked by co-administration of CB2R antagonist/inverse agonist SR144528, and absent in CB2R knockout mice, which indicated these effects are CB2R-dependent [130].

**Table 3:** Synthetic cannabinoids in TBI models

Treatment	Dose	Route	Frequency	Model	Species	Results	References
Arachidonyl-2′-chloroethylamide (ACEA) (CB1R agonist)	1 mg/kg	i.p.	Within 10 min of injury and daily for 6 days (seven doses in total)	CCI (with craniotomy)	Sprague–Dawley rats, males	Treatment attenuated CCI-induced deficits in novel object recognition and Morris water maze performance. Treatment did not alter lesion size after CCI	[141]
HU-910 and HU-914 (CB2R agonists)	0.1–10 mg/kg HU-910 5–10 mg/kg HU-914	i.p.	Single dose 1 h post-injury	Weight-drop mTBI	Sabra mice, males and CB2 KO mice Sprague–Dawley rats, males	Treatment improved NSS over 28 days; inhibited TNFa; increased synaptogenesis; and resulted in partial recovery of corticospinal tract	[130]
JWH-133 or 0-1966 (CB2R agonists) and SR144528 (CB2R antagonist)	1 mg/kg 5 mg/kg 5 mg/kg	i.p.	2 or 18 h post-injury	CCI (with craniotomy)	Wild-type and CB2 KO mice	Treatment reduced sodium fluorescein uptake in CB2 agonist-treated WT mice but not CB2 KO mice; and CB2 KO mice had enhanced TNFa levels	[131]
JWH-133 (CB2R agonist) ± SR144528 (CB2R antagonist)	1.5 mg/kg 3 mg/kg	i.p.	1 h post-injury	CCI (with craniotomy)	Sprague–Dawley rats, males	JWH-133 treatment increased M2 microglia/macrophage polarization via PERK pathway; decreased white matter injury and demyelination; reversed tissue loss; prevented loss of OPCs and OL; restored myelin G-ratio; resulted in fewer unmyelinated axons; restored fractional anisotropy; and improved cerebral blood flow. JWH-133 treatment reduced anxiety-like behavior and improved spatial memory (MWM). Treatment effects were blocked by SR144528	[135]
GP1a (CB2R agonist) ± AM630 (CB2R antagonist)	1–5 mg/kg 5 mg/kg	i.p.	10 min post-injury	CCI (with craniotomy)	C57BL/6 mice, males; and CD1 mice, males	GP1a treatment reduced inflammation; reduced neurovascular injury, biased macrophages to M2; reduced edema; enhanced cerebral blood flow; improved beamwalk and RotaRod performance; and reduced anxiety-like behavior (OFT). AM630 treatment was not beneficial	[136]
0-1966 (CB2R agonist)	5 mg/kg	i.p.	2 or 18 h post-injury for 1 day endpoint, or 2 and 24 h post-injury for 2 day endpoint, and 1, 24, 48, and 72 h post-injury for the 7 day endpoint	CCI (with craniotomy)	C57BL/6 mice, males	Sodium fluorescein uptake peaked 1 day post-CCI. 0-1966 treatment reduced or ameliorated CCI-induced effects, such as increased fluoro-jade labeling, macrophage/microglia counts, and motor impairment	[132]
0-1966 (CB2R agonist)	5 mg/kg	i.p.	1 and 24 h post-injury	CCI (with craniotomy)	C57BL/6 mice, males	CCI impaired motor and exploratory activity. CCI increased cortical edema, substance P, and macrophage/microglia count. 0-1966 treatment reduced all CCI-induced effects	[133]
SMM-189 (CB2R inverse agonist)	6 mg/kg	i.p.	Once daily from day 0 (day of injury) to day 13 (14 doses total)	Blast TBI, blast pressure 50–60 psi above atmospheric pressure applied to left side	C57BL6 mice or EYFP-reporter mice, males	Treatment improved contrast sensitivity; reduced the elevated number of axon bulbs in the optic nerve; reduced axon loss in the left optic nerve; and reduced microglia and GFAP labelling in left optic nerve	[140]
SMM-189 (CB2R inverse agonist)	6 mg/kg	i.p.	Once daily from day 0 (day of injury) to day 13 (14 doses total)	Focal cranial blast injury (mTBI), blast pressure 50–60 psi above atmospheric pressure applied to left side	C57BL6 mice, males	Local field potential coherence was increased in the prefrontal cortex and somatosensory cortex after injury. Treatment restored PFC coherence to control levels. Spike frequency in CA1 was decreased after injury but restored by treatment	[139]
Raloxifene (CB2R inverse agonist and selective estrogen receptor modulator)	5 or 10 mg/kg	i.p.	Once daily from day 0 (day of injury) to day 13 (14 doses total)	Ocular blast injury, blast pressure 50–60 psi above atmospheric pressure applied to left side	C57BL6 mice, males	Treatment improved contrast sensitivity and visual acuity; improved light aversion; rescued ON axon loss in the left eye; improved ipRGC size; and increased melanopsin levels	[137]
Raloxifene (CB2R inverse agonist and selective estrogen receptor modulator)	5 or 10 mg/kg	i.p.	1× daily from day 0 (day of injury) for 4 or 15 daily doses	Single or repeated (5×) ocular blast injury, blast pressure 25 psi above atmospheric pressure	C57BL6 mice, males	Treatment prevented injury-induced light aversion; reduced optic nerve axon loss; improved contrast sensitivity and visual acuity; reduced retinal pathology and optic nerve axon pathology; and biased towards M2 microglia	[138]

Synthetic CB2R agonists preserved BBB integrity in models of TBI and other neuroinflammatory states. The CB2R agonists JWH-133 (1 mg/kg) and 0–1966 (5 mg/kg) and CB2R antagonist (SR144528; 5 mg/kg) were used by Amenta and colleagues [131] to assess the role of CB2R in the neurovascular inflammatory response and BBB changes [131]. Wild-type or CB2R-knockout mice were subjected to the CCI model of TBI, and the CB2R agonists and antagonists were administered 2 or 18 h post-CCI [131]. Injury resulted in increased levels of TNF-α, and this was exacerbated in both CB2R-knockout mice, and in mice treated with the CB2R blocker SR144528 [131]. However, the CB2R agonist JWH-133 did not significantly reduce the heightened TNF-α levels [131]. When BBB permeability was assessed using sodium fluorescein, JWH-133 treatment significantly reduced uptake following CCI, indicating JWH-133 prevented the BBB breach typically observed after injury [131]. This benefit was not observed in CB2R knockout mice, indicating that the beneficial effect of JWH-133 on the BBB was mediated through CB2R-dependent mechanisms [131]. Additional research in the CCI model found that administration of the CB2R agonist 0-1966 reduced Fluoro-Jade C labelling, indicating reduced neurodegeneration after injury [132]. This CB2R agonist had also been found to attenuate CCI-induced edema and substance P elevation (a peptide associated with pain and inflammation) [133].

White matter, including myelin, axons, and oligodendrocytes, is an important therapeutic target for management of TBI symptoms [92]. The vulnerability of white matter to TBI pathology is well established, and dysmyelination and loss of white matter tract integrity likely underlies persistent symptoms after TBI [134]. Therapeutic strategies that protect oligodendrocytes, preserve axonal structure and promote myelination are needed [92]. In a CCI rat model, the CB2R agonist JWH-133 improved multiple measures of white matter pathology. Specifically, injury-induced loss of myelin basic protein and neurofilament-200 were abolished with JWH-133 treatment, with specific effects observed in the corpus callosum, external capsule, cortex, and striatum, to levels comparable to sham-injured rats [135]. Numbers of oligodendrocytes and oligodendrocyte precursor cells were likewise preserved [135], suggesting CB2R signalling supports white matter integrity when faced with injury [135]. Other injury-induced disruptions to white matter included thinner myelin relative to axon diameter and decreased fractional anisotropy (suggested reduced structural integrity), and both of these injury effects were ameliorated with JWH-113 treatment and blocked by CB2R antagonist SR144528 [135]. Treatment with JWH-133 was also associated with a bias towards the protective M2 phenotype of microglia/macrophages, with a ramified appearance and reduced phosphorylated PERK. JWH-133 also reduced clustering of microglia around myelinated fibers and reduced microglia in contact with myelin [135]. Further CB2R-dependent benefits of JWH-133 treatment after CCI injury included improved cerebral blood flow; reduced anxiety-like behavior; and mitigated spatial memory deficits in the Morris Water Maze [135]. Several of these effects, including CB2R-mediated polarization of macrophages to the M2 phenotype; enhanced cerebral blood flow; and improved behavior outcomes, were replicated with administration of another CB2R agonist, GP1a [136].

Interestingly, CB2R inverse agonists have also been assessed in models of blast TBI using raloxifene (also a selective estrogen receptor modulator) and SMM-189, and both treatments were effective in reversing a number of TBI pathologies. Briefly, SMM-189 restored electrophysiological abnormalities after injury and both SMM-189 and raloxifene restored visual acuity, visual contrast sensitivity, and reduced injury pathologies in the retina and optic nerve [137–140]. Overall, these findings support potential therapeutic benefits of CB2R modulation in models of TBI.

While there has been less focus on CB1R alone compared to CB2R, CB1R activation through a synthetic agonist arachidonyl-2′-chloroethylamide (ACEA) administered once daily for 7 days prevented cognitive impairment in the CCI model in both the Morris Water Maze task and Novel Object Recognition task, although treatment did not affect lesion size [141]. In addition to these emerging data, the beneficial effects of endocannabinoids after TBI described previously (see Table 1) are largely dependent on CB1R signaling, suggesting this is a promising target for therapeutic development. In conclusion, exogenous modulation of the endocannabinoid system with phyto- and synthetic cannabinoids is a promising therapeutic strategy, and these data can guide the application of cannabinoids in the treatment of TBI.

### Synthetic cannabinoids in TBI-related conditions

In addition to the previously described research in TBI models, synthetic cannabinoids have also been examined in related neuropathologies, such as stroke and neuronal injury models. In the middle cerebral artery occlusion model, CB1R agonist ACEA treatment reduced infarct volume, neuron apoptosis, and mitochondrial fission [142]. These effects were blocked by the CB1R antagonist AM251, as well as by upregulated dynamin-related protein 1 [142]. The CB1R and CB2R agonist WIN55,212-2 was investigated in a neuron injury model in rats, relevant to HIV and HIV-associated neurocognitive dysfunction caused by the production of neurotoxic and inflammatory proteins, such as GP120 [143]. GP120 toxicity shares characteristics with TBI including glutamate excitotoxicity, elevated intracellular Ca^2+^, oxidative stress, and cell death. The neuronal injury model was produced by injection of GP120 into the hippocampus, and WIN55,212-2 (3 mg/kg) was administered prior to injury and for the following three consecutive days [143]. WIN55,212-2 treatment improved GP120-induced deficits in spatial memory; reduced the number of apoptotic cells; and reduced expression of p38 and JNK mRNA. Treatment also reduced inflammatory mediators and oxidative stress while increasing SOD antioxidant activity. While WIN55,212-2 has activity at both CB1R and CB2R, the beneficial effects were blocked with CB2R inverse agonist AM630, suggesting CB2R was responsible for these effects [143]. To conclude, these studies provide additional support for both CB1R and CB2R-mediated benefits in TBI-relevant pathologies.

## Clinical research of cannabinoids in TBI

### Phytocannabinoids in clinical populations

At present, studies on the effects of cannabinoids in clinical TBI populations are sparse, and no published randomized controlled clinical trials on phytocannabinoids in TBI were located for this review. However, there are a small number of studies on TBI patient populations and cannabis use (Table 4). In one report, 307 patients with physician diagnosed concussions (mTBI) were recruited within 1 week of their injury and followed during recovery via weekly assessments for at least 4 weeks [144]. Participants were surveyed regarding voluntary use of alcohol, cigarettes, and Cannabis before and after injury, and physicians assessed recovery ofcognitive and physical activities. 24.4% of the participants reported using Cannabis regularly prior to their injury, while 14.0% used Cannabis regularly during recovery. None of the substances were associated with improved rate of recovery; however, Cannabis use was associated with a lower symptom severity score in weeks 3 and 4 in unrecovered patients [144]. The increasing ease of access to Cannabis has also permitted the use of toxicology screens to associate the presence or absence of THC in trauma patients upon presentation to hospital with clinical outcomes. Retrospectively, cases that screened positive for THC had a lower mortality rate after TBI [145]. Of the 446 cases included, the overall mortality rate was 9.9%. Overall, 18.4% of the toxicology screens were positive for THC, and a positive screen was associated with a mortality rate of 2.4% compared to 11.5% for THC-negative patients [145]. A similar study found trauma patients that were THC-positive upon presenting to hospital had a shorter median length of stay in hospital and shorter length of stay in intensive care units, although mortality was not affected. In the subset of trauma patients with TBI, THC-positive screening was associated with a shorter hospital stay and fewer ventilator days [146]. In addition, patients with severe TBI who returned a THC-positive screen upon hospital admittance had a lower risk of hemorrhagic stroke compared to those that were THC-negative, though no other differences including thromboembolic outcomes, mortality, or length of hospital stay were found [147]. In United States Military Veterans with a history of mTBI, cannabis use is reported at higher rates compared to the general public and other military veteran populations. The self-reported reasons for cannabis use included management of mTBI-associated symptoms, such as disturbed sleep, pain, and neuropsychiatric symptoms, though cannabis use alone was not sufficient for symptom relief [148]. These are encouraging findings regarding the potential benefits of phytocannabinoids for patients with TBI, though randomized, double-blind, placebo-controlled trials are necessary to confirm these results.

**Table 4:** Cannabinoids in clinical TBI populations

Cannabis use	Dose	Study type	*n*	Injury	Time frame	Results	References
*Phytocannabinoids*							
Self-reported voluntary use of cannabis	n/a	Prospective Observational	307	mTBI; physician diagnosed	Recruitment within 1 week of injury and follow-up at least 4 week post-injury	Cannabis use was not associated with improved recovery rate; cannabis use was associated with reduced symptom severity 3–4 week post-injury	[144]
Self-reported voluntary use of cannabis	n/a	Survey	163	mTBI; United States Military Veterans	Variable (reported use in past month)	Cannabis was used for management of mTBI-related symptoms (sleep, pain, neuropsychiatric symptoms); cannabis use alone was not sufficient for symptom relief	[148]
THC-positive toxicology screen	n/a	Retrospective observational	538	TBI, variable severity	Toxicology screen upon presentation to hospital	Positive THC screen was associated with lower mortality than patients with negative THC screen	[145]
THC-positive toxicology screen	n/a	Retrospective observational	4849	Trauma (severe) with TBI	Toxicology screen upon presentation to hospital	Positive THC screen was associated with shorter hospital stay and shorter duration of ventilator use	[146]
THC-positive toxicology screen	n/a	Retrospective observational	2754	Severe TBI	Toxicology screen upon presentation to hospital	Positive THC screen was associated with lower risk of hemorrhagic stroke; no effect on thromboembolic outcomes, mortality, or length of hospital stay	[147]
*Synthetic cannabinoids*							
Dexanabinol (synthetic cannabinoid derivative; NMDA receptor antagonist)	48 mg or 150 mg (i.v.; single administration)	Phase II RCT (not powered to test efficacy)	67	Severe, closed-head TBI (score of 4–8 on Glasgow Coma Scale)	Within 6 h of injury	Primary Outcome: Treatment was safe and well-tolerated Additional Outcomes: Treatment lowered intracranial pressure; reduced hypotensive episodes; improved cranial perfusion pressure; associated with improved recovery 1 month post-injury	[149]
Dexanabinol (synthetic cannabinoid derivative; NMDA receptor antagonist)	150 mg (i.v.; single administration)	Phase III RCT	861	TBI (score of 2–5 on Glasgow Coma Scale)	Within 6 h of injury	Primary Outcome: Treatment was safe and well-tolerated. No evidence of improved recovery with treatment 6 month post-injury	[150]

### Synthetic cannabinoids in clinical populations

Dexanabinol is a synthetic cannabinoid that has been tested in phase II and phase III clinical trials in TBI patients (Table 4). Dexanabinol is non-psychoactive and is an antagonist at NMDA receptors with anti-oxidant and anti-inflammatory properties [149]. In a phase II study of patients with severe TBI as determined by a score of 4–8 on the Glasgow Coma Scale (and lacking any penetrating head wound, SCI, or major visceral injuries, among other restrictions), a single intravenous injection of Dexanabinol was administered at a dose of 48 mg or 150 mg within 6 h of injury. Treatment was associated with lower intracranial pressure and reduction in the number of hypotensive episodes, suggesting beneficial effects on cerebral edema. This single administration also improved cranial perfusion pressure and was associated with improved recovery 1 month post-injury. Overall, Dexanabinol was found to be safe and well tolerated in patients with severe TBI at the doses examined; yet, despite the benefits seen, the study was not powered to test efficacy and a subsequent phase III trial was required [149]. The phase III clinical trial for Dexanabinol recruited 861 patients across 85 centers and 15 countries [150]. Similar to the phase II study, patients received a single injection of 150 mg of Dexanabinol within 6 h of injury. As with phase II, Dexanabinol was found to be safe and well tolerated, although the benefits observed in the phase II trial were not replicated [150]. The authors noted a limitation of the clinical trial was a lack of data collection regarding the plasma concentration of Dexanabinol, as this may have been altered due to the required administration of fluids during acute care. This could have reduced plasma concentrations and thus altered the efficacy. At present, the data surrounding the therapeutic benefits of cannabinoids in human TBI patient populations remain inconclusive. Further studies are warranted to continue to guide the pursuit of cannabis-based medicine for neurological conditions, such as TBI.

## Conclusions

The endocannabinoid system is increasingly recognized for its physiological role in regulating cellular activity in the brain and endogenous response to adverse events, such as TBI. The ability to modulate this system with endogenous, plant-derived, or synthetic cannabinoids is promising for the development of therapeutic strategies for TBI. Presently, the strongest evidence for neuroprotective properties is seen for compounds containing CBD, or those targeting CB2R, and the effects of THC treatment are less consistent. CBG (and its derivatives) is the most studied minor phytocannabinoid in neurological disease models, while the most evidence for therapeutic benefit from terpenes relates to BCP, although studies are limited overall. The evidence for a modulating, or even synergistic ‘entourage’ effect when cannabinoids are used in combination is still emerging, but full-spectrum plant extracts with a variety of phytocannabinoids may improve the safety and therapeutic profile of cannabinoid medicine.

While the number of studies in preclinical models of TBI has increased with generally positive results, data from clinical populations remain limited. The only cannabinoid-based synthetic pharmaceutical to undergo randomized controlled trials in TBI was Dexanabinol, and it was found not effective; however, the growing literature of cannabinoids in TBI remains promising and further research is warranted. Diseases with complex, multifaceted pathology, such as TBI, may require treatment that is multi-mechanistic, such as whole plant cannabis extracts.

## Data Availability

Not applicable (review article).

## Abbreviations

2-AG: 2-Arachydonoyl glycerol
ACEA: Arachidonyl-2′-chloroethylamide
AEA: N-arachidonylethanolamine (also known as anandamide)
BBB: Blood–brain barrier
BCCAO: Bilateral common carotid artery occlusion
BCP: β-Caryophyllene
Ca2+: Calcium ion
CCI: Controlled cortical impact
CB1R: Cannabinoid receptor 1
CB2R: Cannabinoid receptor 2
CBC: Cannabichromene
CBD: Cannabidiol
CBDA: Cannabidiolic acid
CBDV: Cannabidivarin
CBG: Cannabigerol
CBN: Cannabinol
CNS: Central nervous system
DNA: Deoxyribonucleic acid
EAE: Experimental autoimmune encephalitis
FAAH: Fatty acid amide hydrolase
GABA: Gamma aminobutyric acid
GFAP: Glial fibrillary acid protein
IBA1: Ionized calcium binding adaptor molecule 1
IL-1β: Interleukin 1 beta
MCAO: Middle cerebral artery occlusion
MAGL: Monoacylglycerol lipase
mTBI: Mild traumatic brain injury
NSS: Neurological severity score
PPARγ: Peroxisome proliferator-activated receptor gamma
ROS: Reactive oxygen species
SCI: Spinal cord injury
TBI: Traumatic brain injury
THC: ∆9-Tetrahydrocannabinol
THCV: ∆9-Tetrahydrocannabivarin
THCA: ∆9-Tetrahydrocannabinolic acid
TNF-α: Tumor necrosis factor alpha
TRPV1: Transient receptor potential vanilloid-1
WT: Wild type
